# Complete Mitochondrial Genomes and Evolutionary Insights of Two Commercially Farmed Edible Crickets (*Gryllus bimaculatus* and *Teleogryllus mitratus*) from Thailand

**DOI:** 10.3390/ani16091305

**Published:** 2026-04-23

**Authors:** Pannapak Urairut, Yash Munnalal Gupta, Somjit Homchan

**Affiliations:** Department of Biology, Faculty of Science, Naresuan University, Phitsanulok 65000, Thailand; pannapaku65@nu.ac.th (P.U.); yashmunnalalg@nu.ac.th (Y.M.G.)

**Keywords:** *Gryllus bimaculatus*, *Teleogryllus mitratus*, mitochondrial genome, edible insects, cricket evolution

## Abstract

As global food security challenges intensify, edible crickets are recognized as sustainable protein alternatives; however, genomic resources for commercially important species remain limited, restricting evolutionary inference and the development of robust tools for farm management. We sequenced and assembled the complete mitochondrial genomes of *Gryllus bimaculatus* and provided the first report for *Teleogryllus mitratus*, both derived from commercial farms in Thailand, using high-throughput Illumina sequencing, achieving high coverage depths of 32,391× and 63,258×, respectively. The circular mitochondrial genomes were 15,955 bp and 16,046 bp and exhibited the typical insect mitochondrial gene complement of 37 genes, with a strong AT bias. Selective pressure analyses indicated pervasive purifying selection across all protein-coding genes (PCGs) (ω < 1), while episodic diversifying selection was detected in *cox1*, *cox3*, *cytb*, and *nad5*; additionally, *atp8* displayed a comparatively elevated ω. Codon usage analyses revealed a strong preference for AT-ending codons, with leucine codons showing the highest bias. Phylogenetic analyses using concatenated protein-coding and ribosomal RNA genes recovered well-supported relationships within Gryllidae. These farm-derived mitogenomes provide practical foundations for molecular species authentication, population monitoring, and comparative analyses relevant to breeding and traceability. Furthermore, they provide candidate loci for future investigations into mitochondrial evolutionary dynamics and the potential development of molecular markers for commercial breeding management.

## Simple Summary

The growing interest in edible crickets as sustainable alternatives to traditional livestock highlights a critical need to better understand their genetic makeup for improved farm management. While cricket farming is expanding, our ability to effectively manage breeding and production is often limited by a lack of detailed genetic data for the species raised in commercial environments. In this study, we mapped the complete mitochondrial genomes of two primary commercial cricket species in Thailand: *Gryllus bimaculatus* and *Teleogryllus mitratus.* By identifying the 37 key genes within these genomes, we have established a reliable genetic reference that allows for the accurate identification of these species. These findings provide a baseline genetic reference for species authentication and traceability within the cricket industry. This research establishes a genomic foundation for future studies investigating potential markers associated with performance and adaptation in managed environments.

## 1. Introduction

Edible insects are increasingly recognized as sustainable alternatives to conventional livestock due to their high nutritional value, efficient feed conversion, and lower environmental footprint [1,2]. In Thailand, cricket farming has expanded rapidly and is now a major component of the edible insect industry, providing income for rural communities while supplying domestic and export markets. Two important species, the two-spotted cricket *Gryllus bimaculatus* and *Teleogryllus mitratus*, are widely reared and traded; however, reliable genetic resources remain limited despite their commercial importance [3,4,5,6,7]. Because farm populations may be influenced by founder effects, selective propagation, and the mixing of breeding stocks, providing high-quality genetic reference data is essential for the accurate authentication of commercial species and the monitoring of specific maternal lineages. Such genomic resources provide a necessary baseline for robust traceability across production chains [8] and may support future breeding management by ensuring that processed insect products can be reliably verified against established mitochondrial profiles.

Even with their commercial importance, comprehensive genomic resources for these cricket species remain surprisingly limited. This gap in molecular data constrains the development of diagnostic tools and a preliminary understanding of their evolutionary relationships. Complete mitogenomes provide a necessary foundation for future studies on population genetics and potential investigations into the links between mitochondrial variation and performance traits. Previous phylogenetic studies have relied primarily on partial mitochondrial markers or nuclear genes, approaches that often yield inconsistent results or lack the resolution needed for robust evolutionary inferences [9,10]. Mitogenomes are widely used for phylogenetic inference, population structure analyses, and molecular identification due to their relatively conserved gene content, maternal inheritance, and comparatively rapid mutation rate [11,12].

The mitochondrial genome (mitogenome) is a circular DNA molecule typically ranging from 14 to 20 kb in animals and encodes 13 protein-coding genes (PCGs) involved in oxidative phosphorylation (OXPHOS), 22 tRNAs, two rRNAs, and a non-coding control region that participates in replication and transcription [13,14,15]. In insects, mitogenome sequencing has also contributed to resolving taxonomic ambiguities and understanding evolutionary relationships across diverse lineages [16,17]. For edible insects and farmed species, complete mitogenomes can provide standardized markers for traceability, detect mislabeling or contamination, and provide a basis for future comparative analyses of mitochondrial function [18,19,20].

Despite the growing economic significance of edible crickets, mitogenomic resources remain limited for several species used in commercial farming in Southeast Asia. Previous studies have reported mitogenomes for selected Gryllidae species, but farm-derived reference genomes for Thai production systems are still scarce. Moreover, mitochondrial gene evolution, codon usage bias, and selective pressures have not been sufficiently explored in these farmed crickets, particularly regarding metabolic genes underlying OXPHOS. As farm environments can impose distinct thermal, dietary, and density-related conditions compared to the wild, mitochondrial function may be a component of physiological performance; however, direct evidence of such adaptation requires further integration with ecological and nuclear-genomic data [21].

In this study, we sequenced and annotated the complete mitochondrial genomes of *G. bimaculatus* and provided the first-ever report for *T. mitratus*, using single representative individuals collected from commercial farms in Thailand. We characterized genome organization, nucleotide composition, codon usage patterns, and tRNA structures. We also assessed selective pressures acting on mitochondrial PCGs using codon-based evolutionary models and reconstructed phylogenetic relationships within Gryllidae using concatenated mitochondrial genes. As a resource-based comparative study, this work provides baseline genetic references for commercially important edible crickets in Thailand and supports future efforts in species authentication, traceability, and preliminary genetic monitoring.

## 2. Materials and Methods

### 2.1. Sample Collection and DNA Extraction

Cricket specimens of *G. bimaculatus* and *T. mitratus* were obtained from commercial farms in Phitsanulok and Phayao provinces, Thailand. Fresh specimens were immediately placed in labeled plastic containers and transported to the laboratory, where they were anesthetized before being stored at −20 °C until processing. Total genomic DNA was extracted from the hind leg of a single adult individual for each species using the standard phenol-chloroform method [22], following established protocols for arthropod tissue. DNA quality and concentration were assessed using spectrophotometry and gel electrophoresis before submission to Macrogen, Inc. (Seoul, Republic of Korea) for library preparation and high-throughput sequencing.

### 2.2. Genome Sequencing and Assembly

Paired-end DNA libraries were prepared using the Illumina TruSeq™ Nano DNA Prep Kit (Illumina, Inc., San Diego, CA, USA) and sequenced on an Illumina platform to generate 151 bp paired-end reads. Raw sequencing reads were evaluated for quality using FastQC v0.11.9 [23]. The adapters and low-quality bases were trimmed from raw reads using Trimmomatic v0.39 [24] with the following parameters: LEADING:3, TRAILING:3, SLIDINGWINDOW:4:20, and MINLEN:20. Post-trimming quality was reassessed to ensure successful preprocessing.

High-quality trimmed reads were assembled using NOVOplasty v4.3.1 [25], a de novo organellar genome assembler. We used partial sequences of the cytochrome c oxidase subunit I (*cox1*) gene from closely related cricket species as seed sequences to initiate the assembly process. This seed-based approach specifically targets mitochondrial reads, minimizing the risk of assembling nuclear mitochondrial pseudogenes (NUMTs) or endosymbiont sequences. The *k*-mer length was optimized post hoc through preliminary assembly trials: we selected *k* = 29 for *G. bimaculatus* and *k* = 19 for *T. mitratus*, reflecting species-specific sequence complexity and GC content that modulate optimal *k*-mer sizing. The expected genome size of 16–17 kb, informed by a comparative analysis of published Gryllidae mitogenomes, was used to constrain the assembly search space, reducing erroneous contig formation while accelerating convergence. The average coverage depth for each assembly was reported directly by NOVOplasty based on the number of reads aligned to the final circularized contig.

Complete mitochondrial genomes were annotated using MITOS v1.1.1 [26] with default settings for the invertebrate mitochondrial genetic code. Protein-coding genes, ribosomal RNA genes, transfer RNA genes, and control regions were identified and their boundaries manually refined. We validated annotations by comparing predicted sequences against the NCBI nucleotide database using BLAST v2.17.0 [27]. The annotated mitogenome sequences of *G. bimaculatus* and *T. mitratus* were submitted to the NCBI databases. The GenBank files obtained from NCBI databases were utilized to visualize gene organization and arrangement via Organellar Genome DRAW (OGDRAW) v1.3.1 [28]. The base composition, codon usage, and relative synonymous codon usage (RSCU) of PCGs were analyzed using PhyloSuite v1.2.3 [29]. The selective pressure analysis was performed on a dataset of 24 Gryllidae mitochondrial genomes using the HyPhy software package v2.5.62 via the Datamonkey web server. For both SLAC and BUSTED analyses, the required phylogenetic tree was automatically inferred by the server from the multiple sequence alignment using the Neighbor-Joining (NJ) method, which provided the base topology for estimating ω values and detecting episodic diversifying selection [30,31,32,33,34,35,36]. The secondary structures of tRNAs were inferred using MITOS v1.1.1 on the GALAXY server [26].

### 2.3. Phylogenetic Analysis

To investigate evolutionary relationships within Gryllidae, we compiled a dataset including our two sequenced mitochondrial genomes and 23 previously published Gryllidae mitogenomes retrieved from GenBank [37,38,39,40,41,42,43,44,45,46,47,48,49,50,51,52,53,54,55], while *Ceuthophilus* sp. (accession no. OR551732) and *Gryllotalpa henana* (accession no. NC_071757) served as outgroups based on their established phylogenetic position as sister groups to Gryllidae (see Supplementary Table S1).

For phylogenetic reconstruction, 13 PCGs and two ribosomal RNA genes (12S and 16S rRNA) were extracted from each mitochondrial genome. The sequences were aligned, trimmed, and concatenated using PhyloSuite v1.2.3 [29], with individual alignments of each PCG and rRNA performed using MAFFT v7 [56]. Maximum likelihood (ML) analyses were performed in IQ-TREE v2.2.0 [57], with model selection determined by PartitionFinder2 v2.1.1 [58] under the AIC criterion using the greedy search algorithm, and nodal support was assessed with 1000 ultrafast bootstrap replicates. Bayesian inference (BI) was performed in MrBayes v3.2.7 [59] using two independent Markov Chain Monte Carlo (MCMC) runs of 1,000,000 generations, sampling every 100 generations and discarding the first 25% as burn-in. The resulting phylogenetic trees were visualized and edited in iTOL v7.2.1 [60].

## 3. Results

Assembly of high-depth sequencing reads yielded complete, circular mitochondrial genomes for both species, with coverage depths (32,391× and 63,258× for *G. bimaculatus* and *T. mitratus*, respectively) substantially exceeding thresholds required for confident base-calling and variant detection (GenBank accessions: PP230540 and PP297527). The resulting genomes, at 15,955 bp and 16,046 bp, respectively, align with the expected size distribution for Gryllidae mitogenomes (typically 14–17 kb), suggesting successful capture of complete mitochondrial DNA with minimal loss of genetic material during extraction and sequencing.

**Figure 1:**
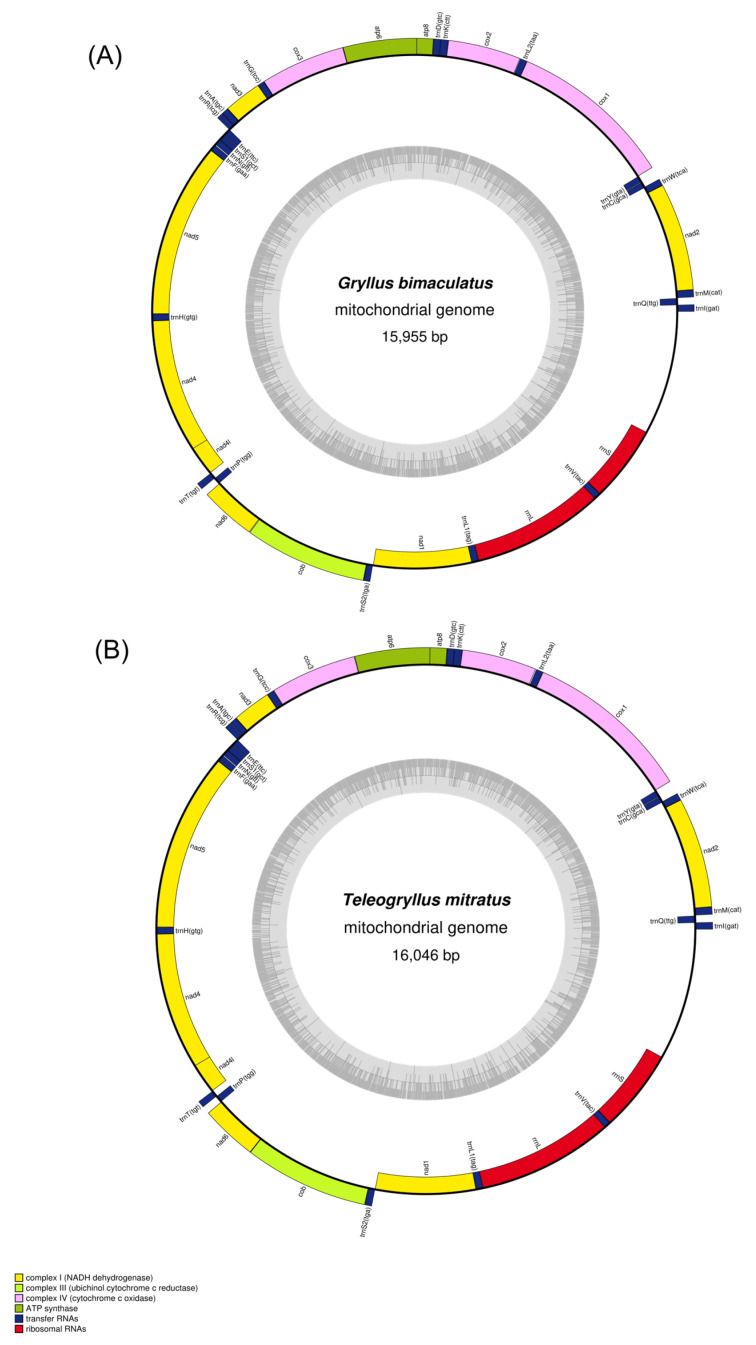
Circular map of the mitochondrial genomes of *G. bimaculatus* (**A**) and *T. mitratus* (**B**). Genes located on the outer circle are transcribed in a clockwise direction (H-strand), while those on the inner circle are transcribed counterclockwise (L-strand). The innermost grey circle represents the GC content across the genome. Genes are color-coded by functional category: yellow (Complex I; NADH dehydrogenase), light green (Complex III; ubiquinol cytochrome c reductase), pink (Complex IV; cytochrome c oxidase), olive green (ATP synthase), dark blue (transfer RNAs), and red (ribosomal RNAs).

As expected from the conserved nature of mitochondrial gene content across animals, both species display the canonical 37-gene architecture characteristic of insects, with composition and organization fully consistent with previously sequenced Gryllidae mitogenomes. Both mitochondrial genomes exhibit the typical insect gene organization, containing 37 genes: 13 protein-coding genes (PCGs), 22 transfer RNA (tRNA) genes, and 2 ribosomal RNA (rRNA) genes, plus a control region, consistent with the metazoan mitochondrial genetic code (Figure 1).

These two mitochondrial genomes exhibit the typical insect gene content and organization, consisting of 13 protein-coding genes (PCGs) *atp6*, *atp8*, *cytb*, *cox1*, *cox2*, *cox3*, *nad1*, *nad2*, *nad3*, *nad4*, *nad4l*, *nad5*, and *nad6* along with 22 transfer RNA (tRNA) genes, including two copies each of **tRNA^Leu^** and tRNA^Ser^, and two ribosomal RNA (rRNA) genes (*rrnS* and *rrnL*).

Gene organization adheres to the asymmetric strand distribution typical of insects, with both species encoding 4 PCGs and 11 tRNAs on the minority (N) strand alongside both rRNAs, while 9 PCGs and 11 tRNAs occupy the majority (J) strand. This division reflects underlying constraints related to concurrent transcription and replication, wherein genes on opposite strands experience different mutational pressures [50]. At the codon level, most PCGs initiate with standard ATN start codons; however, *cox1* and *nad1* deviate from this pattern, employing alternative start codons (CAA and TTG in *G. bimaculatus*; CGA and TTG in *T. mitratus*). Such non-canonical initiation codons, increasingly documented in Orthoptera [50,61], likely reflect the permissive nature of mitochondrial translation systems and may reduce ribosome stalling relative to standard AUG initiation in the AT-rich mitochondrial genome. Termination patterns showed predominant use of the complete TAA or TAG stop codon. However, several genes terminated with incomplete stop codons. Specifically, the *nad4* gene in *T. mitratus* used an incomplete ‘TA’ stop codon, while *cox1* and *cox2* in both species, along with *cox3*, *nad5*, and *cytb* in *T. mitratus*, terminated with a single ‘T’ (Supplementary Table S2). Such incomplete stop codons are a common feature in arthropod mitochondrial genomes and are typically completed to TAA via post-transcriptional polyadenylation [62,63].

Nucleotide composition analysis revealed a strong AT bias in both species, with adenine and thymine comprising 74.0% and 73.4% of total nucleotides in *G. bimaculatus* and *T. mitratus*, respectively. This AT-rich composition varied among genomic regions: protein-coding genes showed the highest AT content (73.4–74.9%), followed by rRNAs (73.5–74.9%) and tRNAs (75.5–76.0%). Strand asymmetry analysis using AT-skew and GC-skew values revealed distinct patterns across genomic regions. The complete mitochondrial genomes exhibited positive AT-skew (0.095 in *G. bimaculatus*, 0.082 in *T. mitratus*) and negative GC-skew (−0.31 and −0.28, respectively), indicating strand-specific mutational biases. Notably, PCGs displayed contrasting patterns with negative AT-skew (−0.138 to −0.141) but near-neutral GC-skew, while rRNAs showed negative AT-skew but strongly positive GC-skew (0.365–0.398). Transfer RNAs exhibited intermediate patterns with near-neutral AT-skew and moderately positive GC-skew values (Table 1).

**Figure 2:**
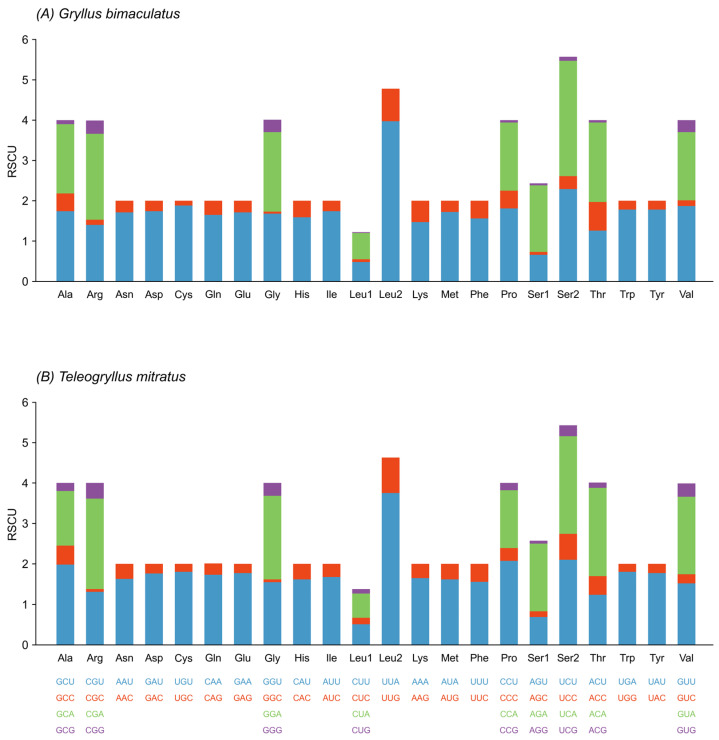
Relative synonymous codon usage (RSCU) of protein-coding genes in the complete mitochondrial genome of *G. bimaculatus* (**A**) and *T. mitratus* (**B**). The RSCU values are color-coded based on the codon below the amino acid labels.

Analysis of relative synonymous codon usage (RSCU) across all 13 PCGs revealed a strong codon bias toward A/T-ending codons in both species (Figure 2 and Supplementary Table S3). Of the 62 sense codons analyzed, both species showed a marked preference for NNU and NNA codons, with UUA (Leu2) displaying the highest RSCU values (3.97 in *G. bimaculatus*, 3.75 in *T. mitratus*). In contrast, the RSCU values for CUG (Leu1), which end with G and C, were relatively low. Notably, Leu2 and Ser2 consistently had the highest RSCU values in both species.

**Figure 3:**
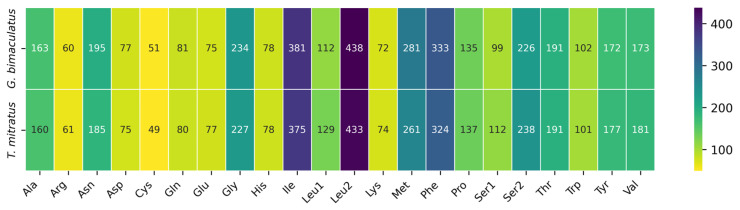
Codon usage distribution and comparison in the mitochondrial genomes of *G. bimaculatus* and *T. mitratus*.

Amino acid composition analysis revealed highly conserved usage patterns between *G. bimaculatus* and *T. mitratus* (Figure 3 and Supplementary Table S3). In both species, leucine (UUR and CUN codons) was the most frequently used amino acid, with a combined count of over 800 residues, followed by phenylalanine. Conversely, cysteine and tryptophan were the least utilized. While minor interspecific variations in specific amino acid counts were observed, such as slightly higher serine 2 and proline in *T. mitratus*. These differences represent a negligible proportion of the total protein-coding content. Overall, the amino acid distribution remains remarkably stable across both mitogenomes.

**Figure 4:**
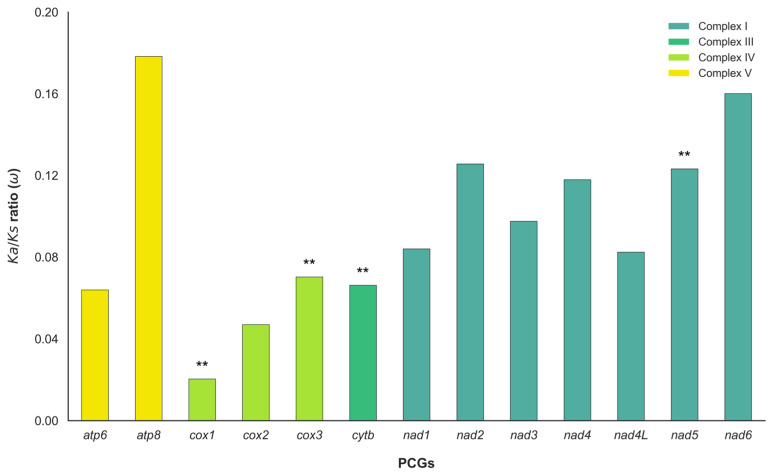
Evolutionary rates of mitochondrial protein-coding genes (PCGs). The bar chart represents the Ka/Ks ratio (ω) for each gene, categorized by respiratory complex (Complexes I, III, IV, and V). Asterisks (**) denote significant likelihood ratio tests (*p* < 0.05).

Differential selective pressures among mitochondrial genes have important implications for understanding metabolic evolution and identifying candidate genes for functional studies. To assess these pressures, we employed codon-based substitution rate analyses. The ω ratios for all PCGs were below 1, indicating that mitochondrial genes are predominantly evolving under pervasive purifying selection. Consistent with this, the BUSTED analysis detected gene-wide evidence of episodic diversifying selection in *cox1*, *cox3*, *cytb*, and *nad5*, as indicated by significant likelihood ratio tests (*p* < 0.05; marked by asterisks). While these results indicate non-neutral evolution, they represent candidate loci for further investigation rather than definitive proof of functional adaptation to commercial rearing. Among the PCGs, *atp8* showed a relatively elevated ω, consistent with weaker functional constraints and a faster evolutionary rate compared with other mitochondrial genes. Overall, these results demonstrate heterogeneity in selective regimes among mitochondrial genes and respiratory chain complexes, with pervasive purifying selection dominating the mitochondrial genome despite episodic diversifying selection acting on specific genes (Figure 4 and Supplementary Table S4).

**Figure 5:**
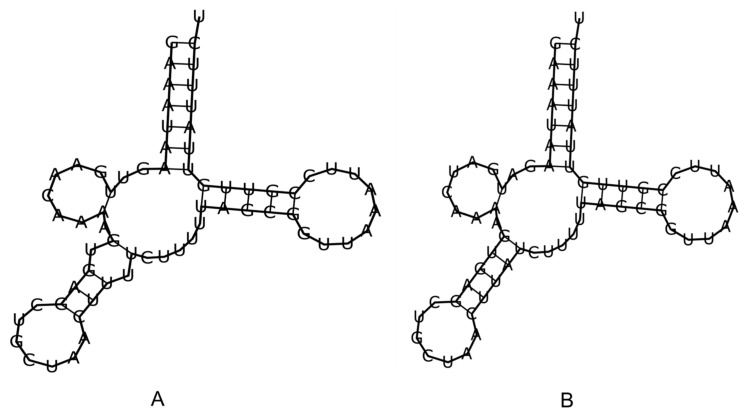
Secondary structures of tRNA^Ser1^ found in the mitogenome of *G. bimaculatus* (**A**) and *T. mitratus* (**B**) that lack a dihydrouridine (DHU) arm.

The mitochondrial genomes of both *G. bimaculatus* and *T. mitratus* encode a standard set of 22 transfer RNAs (tRNAs) (Supplementary Figures S1 and S2). Most tRNAs in both species exhibit the typical cloverleaf secondary structure, with the exception of *trnS1* (GCU), which lacks a functional dihydrouridine (DHU) arm in both taxa (Figure 5). Comparative analysis reveals that *T. mitratus* possesses more compact tRNA structures than *G. bimaculatus.* Specifically, significant truncation of the T-arm was observed in *trnT* and *trnW* of *T. mitratus*, while the D-arm of *trnS2* in *T. mitratus* showed a more pronounced reduction compared to the relatively stable cloverleaf form found in *G. bimaculatus.* Further information is shown in Supplementary Table S5.

Both species contain complete large and small ribosomal RNA subunits positioned in the typical arthropod arrangement: lrRNA between tRNA^Leu^ and tRNA^Val^, and srRNA between tRNA^Val^ and the control region. The lrRNA genes measured 1313 bp (*G. bimaculatus*) and 1315 bp (*T. mitratus*), while srRNA genes spanned 762 bp and 808 bp, respectively. These size variations fall within the normal range for Gryllidae species and are consistent with minor indel events in non-critical structural regions.

**Figure 6:**
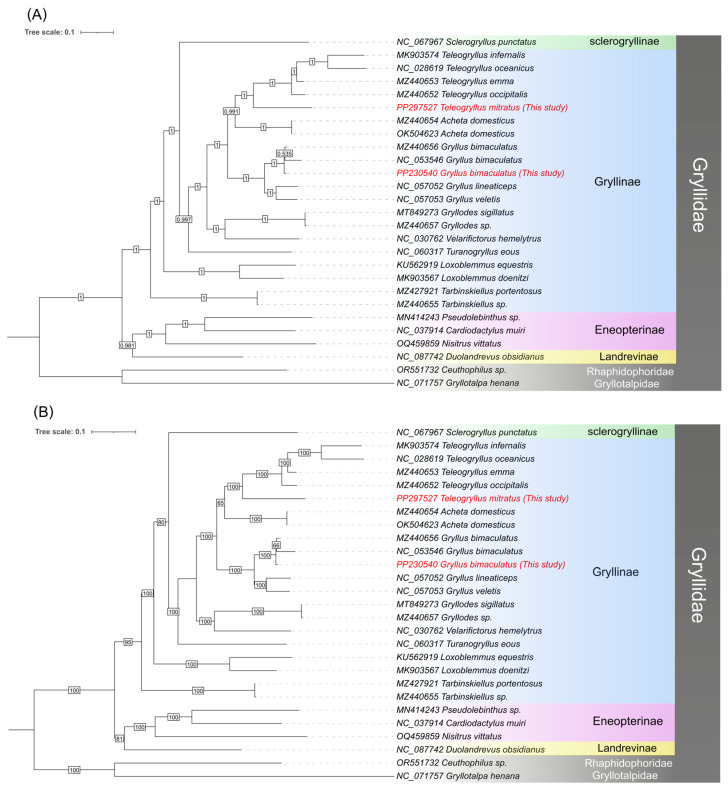
Phylogenetic relationships among 24 Gryllidae taxa based on mitochondrial genome sequences. Phylogenetic tree inferred from Bayesian inference (BI) (**A**) and maximum likelihood (ML) (**B**) analyses. Numbers at the nodes represent Bayesian posterior probabilities and ML bootstrap values (%), respectively. Colored blocks indicate subfamily-level classifications within the Gryllidae family. The two species newly sequenced in this study are highlighted in red. *Ceuthophilus* sp. (Rhaphidophoridae) and *Gryllotalpa henana* (Gryllotalpidae) were used as outgroups.

The phylogenetic analyses recovered well-supported clades consistent with established genera within the family Gryllidae. The phylogenetic trees generated by both ML and BI methods exhibited identical topologies (topological congruence), differing only in minor branch lengths. All three focal genera, *Gryllus*, *Teleogryllus*, and *Tarbinskiellus*, were recovered as monophyletic groups with high statistical support (ML bootstrap ≥ 95%; BI posterior probability ≥ 0.95). The two newly sequenced species were placed firmly within their respective genera: *G. bimaculatus* clustered with other *Gryllus* species, while *T. mitratus* grouped with *T. emma* and *T. infernalis*. The tree was rooted using *Ceuthophilus* sp. and *G. henana* as outgroups, which were clearly separated from the ingroup taxa, providing additional confidence in the inferred topology. The congruent results from both ML and BI analyses, combined with consistently high support values, demonstrate the effectiveness of mitochondrial genome data for resolving phylogenetic relationships within edible cricket lineages (Figure 6 and Supplementary Table S6).

## 4. Discussion

This study presents the complete mitochondrial genomes of *G. bimaculatus* and provides the first report of the complete mitogenome for *T. mitratus* from commercial farms in Thailand, substantially expanding the genomic infrastructure for one of Southeast Asia’s most economically significant edible insect industries. Beyond documenting genome sequences, our comparative analyses reveal heterogeneous evolutionary dynamics across mitochondrial genes, with implications for understanding the selective pressures acting on the mitogenome across the Gryllidae lineage. Such insights provide a genomic baseline to eventually explore how mitochondrial function might relate to different ecological or managed environments in future studies. The overall gene content and organization of both mitogenomes conform to the conserved arthropod pattern, consistent with strong functional constraints on mitochondrial genome architecture across Gryllidae [40,46,53]. However, the presence of non-canonical start codons (e.g., CAA, CGA, and TTG) and incomplete stop codons (T or TA) represents a common feature in insect mitochondrial genomes that likely reflects the specialized nature of mitochondrial translation systems [50,61,64,65,66].

Comparison with previously reported *G. bimaculatus* mitogenomes (accession numbers: NC_053546 and MZ440656) [38,40] confirmed overall structural conservation, with identical gene organization and comparable AT-richness. However, a notable difference was observed in the D-loop repeat array between our newly assembled mitogenome (PP230540) from Phitsanulok, Thailand, and the published sequence MZ440656, which was sampled from Hanoi, Vietnam. Our assembly contains two complete 219 bp repeats in tandem, followed by a 195 bp partial copy, whereas MZ440656 has only one complete repeat followed by the same partial copy. The extra repeat is supported by SRA BLAST analyses of short-read datasets (DRX261895 and DRX261894) and a long-read dataset (DRX261898), alongside an exceptional read depth across the region (32,391×). While these independent data sources provide strong support for the biological reality of this tandem repeat, we acknowledge the inherent challenges in assembling highly repetitive non-coding regions.

Such D-loop repeat polymorphism may reflect geographic differentiation between regional Indochinese populations, a phenomenon consistent with reported insertions/deletions in the control regions of other Thai crickets, such as *Acheta domesticus* [41]. Nevertheless, the possibility of intra-individual heteroplasmy should be acknowledged, as tandem repeats in mtDNA are susceptible to slipped-strand mispairing. Consequently, this D-loop variation serves as a substantial marker class; for instance, diagnostic assays targeting these repeat junctions could be developed for efficient maternal-lineage tracking and high-resolution intraspecific differentiation without the need for full mitogenome sequencing. Future validation through Sanger sequencing or targeted long-read sequencing of this specific junction will be beneficial to definitively rule out potential assembly artifacts.

Both species exhibit pronounced AT richness, with genome-wide AT content exceeding 73–74%, consistent with broad insect patterns and Gryllidae-specific comparative work [50,67,68]. The combination of positive AT-skew and negative GC-skew indicates strand-asymmetric substitution pressures that are plausibly linked to mutational biases associated with replication and transcription [69]. Crucially, this compositional landscape provides the most parsimonious explanation for the observed codon-usage bias toward A/U-ending codons and the high representation of leucine and phenylalanine residues, patterns typical of orthopteran mitogenomes [46,70,71]. While translational selection could theoretically contribute to maintaining efficiency in OXPHOS genes, the observed codon bias is primarily consistent with the underlying nucleotide composition, and further empirical data are required to evaluate any secondary selective contribution [16,17,72].

The selective pressure analyses revealed pronounced heterogeneity in evolutionary dynamics among mitochondrial protein-coding genes (Figure 4). Although pervasive purifying selection predominated across the mitogenome, as reflected by mean ω values below 0.2 for all PCGs, several genes exhibited signatures of episodic diversifying selection. Specifically, *cox1*, *cox3*, *cytb*, and *nad5* showed significant gene-wide evidence of positive selection (BUSTED, *p* < 0.05; indicated by ** in Figure 4). When analyzing these results by respiratory complex, we observed that while selective constraints were generally uniform, genes within Complex IV (the *cox* family) displayed the lowest ω values, indicating stronger functional conservation. In contrast, specific subunits in Complex I (*nad5*) and Complex III (*cytb*) may represent candidate loci for future investigations of mitochondrial evolutionary dynamics within Gryllidae, potentially reflecting long-term evolutionary adjustments rather than immediate responses to specific rearing conditions [73,74].

Despite evidence of episodic diversifying evolution in certain respiratory genes, the high overall nucleotide identity (98–99%) with previously reported *G. bimaculatus* sequences suggests that the fundamental architecture of the mitogenome remains highly conserved across different populations. This level of conservation supports the use of these genomes for reliable species authentication, while the identified variations provide a potential resolution for distinguishing commercial breeding lines. The intermittent nature of positive selection suggests that these pressures may fluctuate over time rather than remain constant, with only certain lineages or time periods experiencing directional selection on electron transport chain (ETC) function [75]. Future work incorporating functional data on thermal tolerance, metabolic rate, and feed efficiency across natural and farmed populations will be essential to test whether positive selection signatures correlate with physiological performance differences.

In contrast, *atp8* (Complex V) displayed a relatively elevated ω compared to other PCGs, indicating substantially weaker functional constraints and a faster evolutionary rate. This pattern, consistent with observations in other orthopterans [40,42,76], likely reflects the reduced functional stringency of ATP synthase subunit 8. As a small protein of approximately 49 amino acids in crickets, *atp8* may possess fewer structural requirements compared to the larger, core catalytic subunits. While this elevation typically points toward relaxed functional constraints, any potential for directional functional adaptation remains speculative without further biochemical and physiological evidence [77].

The secondary structures of mitochondrial tRNAs in both *G. bimaculatus* and *T. mitratus* conform to the classical cloverleaf model, comprising the acceptor stem, dihydrouridine (DHU) arm, anticodon arm, variable loop, and TΨC arm. The sole exception is *trnS1* (AGN), which notably lacks the DHU arm, a feature widely observed across metazoan mitochondrial genomes, particularly in insects [67,78,79,80], and is likely compensated by specialized mitochondrial elongation factors during translation. However, the more extensive truncation of T-arms and D-arms in *T. mitratus* suggests a more derived state of genome minimization compared to *G. bimaculatus.* Notably, this recurrent structural reduction is widely treated as a lineage-consistent feature of metazoan mitochondrial tRNAs rather than a loss of function; its presence here is, therefore, consistent with accurate annotation and expected mitochondrial tRNA evolution [68,78].

The robust phylogenetic resolution achieved here using mitochondrial data alone represents a considerable strength for applied purposes, such as species authentication and population tracking; however, it also highlights an important limitation of mitochondrial-only phylogenetics: the single-locus nature of mtDNA means that the recovered topology, while well-supported, represents the evolutionary history of maternal lineages specifically [81,82]. In farmed populations, where animals may be crossed among diverse maternal and paternal genetic backgrounds [3,83], the mitochondrial phylogeny may not fully capture the nuclear-genomic structure or the extent of admixture among populations. Nonetheless, the monophyly of *Gryllus* and *Teleogryllus* and the strong support for species-level relationships provide confidence that these genera represent distinct evolutionary lineages and not unrecognized species complexes, an important consideration given the economic value of reliable species identification.

It is important to acknowledge the limitations of the current study. As our analysis is based on single representative individuals per species, these data do not assess intraspecific mitochondrial diversity, variation among different farms, or differences between farmed and wild populations. Furthermore, without correlated phenotypic data from a larger population, the identified variations cannot be directly linked to production traits at this stage. These findings should, therefore, be viewed as a high-quality genomic foundation for future large-scale population studies and functional investigations.

The genomic resources presented here enable several practical innovations in cricket agriculture. First, the complete genome sequences permit the design of species-specific molecular assays (real-time PCR, high-resolution melt analysis) for rapid, accurate species identification [84], a critical quality-control metric given the market premium for *G. bimaculatus* and *T. mitratus* relative to less-valued species. Second, the identification of polymorphisms in the mitochondrial D-loop provides a foundation for developing population-specific markers to track maternal lineages; our assembly reveals copy-number polymorphism in repeat arrays not present in previously published genomes. In the future, these markers could potentially be integrated into studies aiming to associate mitochondrial haplotypes with traits such as growth rate or fecundity. Third, the genes identified under positive selection represent candidate loci for future research into mitochondrial evolutionary dynamics. These resources provide a genomic foundation to eventually test hypotheses regarding how mitochondrial variation might correlate with physiological traits, although such links remain speculative at present. Lastly, an understudied application involves quality assurance in cricket farming supply chains. Because crickets are marketed both alive and as processed products (e.g., whole-insect meals, protein powders), molecular markers enabling traceability from farm to consumer could authenticate provenance claims and detect species substitution or contamination with wild insects or unrelated species [18,19,20]. Mitochondrial barcoding, leveraging the complete genomes presented here, offers a rapid, cost-effective pathway to implement such traceability, with particular relevance for premium markets in Thailand and the EU, where food authentication and supply-chain transparency are increasingly demanded by consumers and regulators.

## 5. Conclusions

This study provides complete mitogenomes for *G. bimaculatus* and *T. mitratus*, establishing baseline genomic references for commercially farmed edible crickets in Thailand. Our findings reveal that while purifying selection predominantly preserves OXPHOS architecture, signatures of episodic diversifying selection were detected in *cox1*, *cox3*, *cytb*, and *nad5*. These genes represent candidate loci for future research into mitochondrial evolutionary dynamics; however, whether these patterns are linked to functional adaptation or environmental factors remains to be validated. These resources directly facilitate molecular traceability and species authentication within the cricket supply chain. Future research incorporating broader population sampling and phenotypic data will be essential to test the functional significance of these mitochondrial variations and to support the long-term sustainability of insect agriculture.

## Figures and Tables

**Table 1:** Nucleotide composition of two mitogenomes (*G. bimaculatus* and *T. mitratus*).

Species	Regions	Length (bp)	A (%)	T (%)	C (%)	G (%)	AT (%)	GC (%)	AT-Skew	GC-Skew
*G. bimaculatus*	Mitogenomes	15,955	40.5	33.5	16.9	9	74	25.9	0.095	−0.31
	PCGs	11,136	31.1	41.7	13.3	13.3	73.4	26.6	−0.138	0.001
	tRNAs	1448	37.6	38.4	9.5	14.4	76	23.9	−0.01	0.205
	rRNAs	2121	33.7	41.2	8	17.1	74.9	25.1	−0.1	0.365
*T. mitratus*	Mitogenomes	16,046	39.7	33.7	17	9.6	73.4	26.6	0.0821	−0.28
	PCGs	11,196	31.2	41.4	13.8	13.7	72.6	27.5	−0.141	−0.004
	tRNAs	1452	38.6	36.9	9.8	14.7	75.5	24.5	0.022	0.202
	rRNAs	2133	31.9	41.6	8	18.5	73.5	26.5	−0.131	0.398

## Data Availability

The assembled mitochondrial genome sequence that supports this study is available at NCBI (https://www.ncbi.nlm.nih.gov/). Nucleotide sequence data reported are available in the DDBJ/ENA/GenBank databases under the accession numbers PP230540 (*G. bimaculatus*) and PP297527 (*T. mitratus*).

## Supplementary Materials

The following supporting information can be downloaded at: https://www.mdpi.com/article/10.3390/ani16091305/s1. Figure S1: Secondary structures of 22 tRNAs found in the mitogenome of *G. bimaculatus.* Most structures follow a standard cloverleaf model, with the notable exception of *trnS1*(GCU), which lacks the DHU arm. For a detailed side-by-side structural comparison and identification of conserved or variable regions relative to *T. mitratus*, refer to Supplementary Table S5; Figure S2: Secondary structures of 22 tRNAs found in the mitogenome of *T. mitratus.* Similar to *G. bimaculatus*, the *trnS1*(GCU) in this species also exhibits a missing DHU arm. Specific variations in stem lengths, loop sizes, and other structural differences compared to *G. bimaculatus* are summarized in Supplementary Table S5; Table S1: GenBank accession number of species and taxonomic information for the phylogenetic analysis; Table S2: List of mitogenome configurations of the cricket species; Table S3: Frequency and RSCU values of codons in PCGs in the mitogenomes; Table S4: Ka/Ks ratio of 13 protein-coding genes in 24 species of Gryllidae; Table S5: Comparative analysis of the secondary structures of 22 tRNAs between *G. bimaculatus* and *T. mitratus*; Table S6: Results of best fit models for each alignment partition by PartitionFinder2.
