# Supplementary material for: Complete Mitochondrial Genomes and Evolutionary Insights of Two Commercially Farmed Edible Crickets (Gryllus bimaculatus and Teleogryllus mitratus) from Thailand
Source: Animals (Basel). 2026 Apr 23;16(9):1305. doi: 10.3390/ani16091305 (PMC13163110; doi:10.3390/ani16091305)
Supplement: Supplementary file 1 [file animals-16-01305-s001.zip › animals-4236102-supplementary/Figure captions (supplementary).pdf]

Figure captions (Supplementary)

Figure S1. Secondary structures of 22 tRNAs found in the mitogenome of *G. bimaculatus*. Most structures follow a standard cloverleaf model, with the notable exception of *trnS1*(GCU) which lacks the DHU arm. For a detailed side-by-side structural comparison and identification of conserved or variable regions relative to T. mitratus, please refer to **Supplementary Table S5.**

Figure S2. Secondary structures of 22 tRNAs found in the mitogenome of *T. mitratus*. Similar to *G. bimaculatus*, the *trnS1*(GCU) in this species also exhibits a missing DHU arm. Specific variations in stem lengths, loop sizes, and other structural differences compared to *G. bimaculatus* are summarized in **Supplementary Table S5.**
