# Supplementary material for: Complete Mitochondrial Genomes and Evolutionary Insights of Two Commercially Farmed Edible Crickets (Gryllus bimaculatus and Teleogryllus mitratus) from Thailand
Source: Animals (Basel). 2026 Apr 23;16(9):1305. doi: 10.3390/ani16091305 (PMC13163110; doi:10.3390/ani16091305)
Supplement: Supplementary file 1 [file animals-16-01305-s001.zip › animals-4236102-supplementary/Supplementary tables_Animals.pdf]

Supplementary Table S1. GenBank accession number of species and taxonomic information for the phylogenetic analysis.

Superfamily	Family	Subfamily	Species	Accession	References
Grylloidea	Gryllidae	Gryllinae	*Gryllus bimaculatus*	PP230540	**This study**
Grylloidea	Gryllidae	Gryllinae	*Teleogryllus mitratus*	PP297527	**This study**
Grylloidea	Gryllidae	Gryllinae	*Gryllus bimaculatus*	MZ440656	(Sanno et al., 2021)
Grylloidea	Gryllidae	Gryllinae	*Gryllus bimaculatus*	NC_053546	(Park et al., 2021)
Grylloidea	Gryllidae	Gryllinae	*Gryllus veletis*	NC_057053	(Torson et al., 2022)
Grylloidea	Gryllidae	Gryllinae	*Gryllus lineaticeps*	NC_057052	(Torson et al., 2022)
Grylloidea	Gryllidae	Gryllinae	*Teleogryllus emma*	MZ440653	(Sanno et al., 2021)
Grylloidea	Gryllidae	Gryllinae	*Teleogryllus occipitalis*	MZ440652	(Sanno et al., 2021)
Grylloidea	Gryllidae	Gryllinae	*Acheta domesticus*	OK504623	(Homchan et al., 2024)
Grylloidea	Gryllidae	Gryllinae	*Teleogryllus infernalis*	MK903574	(Chang et al., 2020)
Grylloidea	Gryllidae	Gryllinae	*Acheta domesticus*	MZ440654	(Sanno et al., 2021)
Grylloidea	Gryllidae	Gryllinae	*Velarifictorus hemelytrus*	NC_030762	(Yang et al., 2016)
Grylloidea	Gryllidae	Gryllinae	*Gryllodes sigillatus*	MT849273	(Yuan et al., 2021)
Grylloidea	Gryllidae	Gryllinae	*Gryllodes* sp.	MZ440657	(Sanno et al., 2021)
Grylloidea	Gryllidae	Gryllinae	*Turanogryllus eous*	NC_060317	(Ma et al., 2019)
Grylloidea	Gryllidae	Gryllinae	*Loxoblemmus doenitzi*	MK903567	(Chang et al., 2020)
Grylloidea	Gryllidae	Sclerogryllinae	*Sclerogryllus punctatus*	NC_067967	(Yu et al., 2022)
Grylloidea	Gryllidae	Gryllinae	*Tarbinskiellus* sp.	MZ440655	(Sanno et al., 2021)
Grylloidea	Gryllidae	Gryllinae	*Tarbinskiellus portentosus*	MZ427921	(Wang et al., 2022)
Grylloidea	Gryllidae	Gryllinae	*Loxoblemmus equestris*	KU562919	(Yang et al., 2016)
Grylloidea	Gryllidae	Landrevinae	*Duolandrevus obsidianus*	NC_087742	(Chen et al., 2019), (Xu et al., 2021)
Grylloidea	Gryllidae	Eneopterinae	*Nisitrus vittatus*	OQ459859	(Dong et al., 2024)
Grylloidea	Gryllidae	Eneopterinae	*Pseudolebinthus* sp.	MN414243	(Salazar et al., 2020)
Grylloidea	Gryllidae	Gryllinae	*Teleogryllus oceanicus*	NC_028619	(Zhou et al., 2017)
Grylloidea	Gryllidae	Eneopterinae	*Cardiodactylus muiri*	NC_037914	(Dong et al., 2017)
Tettigoniidea	Rhaphidophoroidea	Rhaphidophoridae	*Ceuthophilus* sp.	OR551732	(Dowle et al., 2024)
Gryllotalpoidea	Gryllotalpidae	Gryllotalpinae	*Gryllotalpa henana*	NC_071757	(Ma & Miao, 2022)

Supplementary Table S2. List of mitogenomes configurations of the cricket species.

G. bimaculatus (1-15,955)						
Gene	Direction	Location	Size (bp)	Start codon	Stop codon	INC
trnI	J	1-67	67	-	-	0
trnQ	N	65-133	69	-	-	-3
trnM	J	136-204	69	-	-	2
nad2	J	205-1,221	1,017	ATT	TAA	0
trnW	J	1,220-1,286	67	-	-	-2
trnC	N	1,279-1,340	62	-	-	-8
trnY	N	1,343-1,407	65	-	-	2
cox1	J	1,409-2,939	1,531	CAA	T	1
trnL2	J	2,940-3,003	64	-	-	0
cox2	J	3,006-3,681	676	ATG	T	2
trnK	J	3,682-3,751	70	-	-	0
trnD	J	3,752-3,816	65	-	-	0
atp8	J	3,817-3,972	156	ATT	TAA	0
atp6	J	3,966-4,649	684	ATG	TAA	-7
cox3	J	4,649-5,437	789	ATG	TAA	-1
trnG	J	5,437-5,499	63	-	-	-1
nad3	J	5,500-5,853	354	ATT	TAG	0
trnA	J	5,853-5,916	64	-	-	-1
trnR	J	5,917-5,978	62	-	-	0
trnE	N	5,977-6,043	67	-	-	-2
trnS1	N	6,045-6,111	67	-	-	1
trnN	N	6,112-6,177	66	-	-	0
trnF	N	6,184-6,248	65	-	-	6
nad5	N	6,248-7,999	1,752	ATT	TAA	-1
trnH	N	8,000-8,067	68	-	-	0
nad4	N	8,067-9,410	1,344	ATG	TAG	-1
nad4L	N	9,404-9,700	297	ATG	TAA	-7
trnT	J	9,704-9,768	65	-	-	3
trnP	N	9,769-9,834	66	-	-	0
nad6	J	9,837-10,358	522	ATT	TAA	2
cytb	J	10,362-11,498	1,137	ATG	TAA	3
trnS2	J	11,498-11,559	62	-	-	-1
nad1	N	11,562-12,524	963	TTG	TAA	2
trnL1	N	12,525-12,590	66	-	-	0
rrnL	N	12,591-13,903	1,313	-	-	0
trnV	N	13,886-13,954	69	-	-	-18
rrnS	N	13,954-14,715	762	-	-	-1
D-loop	J	14,716-15,955	1,240	-	-	0

Supplementary Table S2. List of mitogenomes configurations of the cricket species. (cont.)

T. mitratus (1-16,046)						
Gene	Direction	Location	Size (bp)	Start codon	Stop codon	INC
trnI	J	1-67	67	-	-	0
trnQ	N	65-133	69	-	-	-3
trnM	J	137-205	69	-	-	3
nad2	J	206-1219	1,014	ATT	TAA	0
trnW	J	1218-1285	68	-	-	-2
trnC	N	1278-1338	61	-	-	-8
trnY	N	1341-1405	65	-	-	2
cox1	J	1410-2937	1,528	CGA	T	4
trnL2	J	2938-3001	64	-	-	0
cox2	J	3007-3685	679	ATG	T	5
trnK	J	3686-3755	70	-	-	0
trnD	J	3756-3820	65	-	-	0
atp8	J	3821-3976	156	ATT	TAA	0
atp6	J	3970-4653	684	ATG	TAA	-7
cox3	J	4655-5441	787	ATG	T	1
trnG	J	5442-5505	64	-	-	0
nad3	J	5506-5859	354	ATC	TAG	0
trnA	J	5859-5924	66	-	-	-1
trnR	J	5926-5987	62	-	-	1
trnE	N	5986-6052	67	-	-	-2
trnS1	N	6054-6120	67	-	-	1
trnN	N	6121-6186	66	-	-	0
trnF	N	6192-6259	68	-	-	5
nad5	N	6260-8000	1,741	ATT	T	0
trnH	N	8001-8065	65	-	-	0
nad4	N	8066-9408	1,343	ATG	TA	0
nad4L	N	9402-9698	297	ATG	TAA	-7
trnT	J	9701-9764	64	-	-	2
trnP	N	9765-9831	67	-	-	0
nad6	J	9834-10352	519	ATT	TAA	2
cytb	J	10352-11486	1,135	ATG	T	-1
trnS2	J	11487-11550	64	-	-	0
nad1	N	11549-12514	966	TTG	TAA	-2
trnL1	N	12515-12580	66	-	-	0
rrnL	N	12581-13905	1,325	-	-	0
trnV	N	13888-13955	68	-	-	-18
rrnS	N	13955-14762	808	-	-	-1
D-loop	J	14763-16046	1,284	-	-	0

Supplementary Table S3. Frequency and RSCU values of codon in PCGs in the mitogenomes.

G. bimaculatus															
**Amino acid**	**Codon**	**Count**	**RSCU**	**Amino acid**	**Codon**	**Count**	**RSCU**	**Amino acid**	**Codon**	**Count**	**RSCU**	**Amino acid**	**Codon**	**Count**	**RSCU**
Phe	UUU	259	1.56	Ser2	UCU	93	2.29	Tyr	UAU	153	1.78	Cys	UGU	48	1.88
	UUC	74	0.44		UCC	13	0.32		UAC	19	0.22		UGC	3	0.12
Leu2	UUA	364	3.97		UCA	116	2.86	Stop	UAA (*)	-	-	Trp	UGA	91	1.78
	UUG	74	0.81		UCG	4	0.1		UAG (*)	-	-		UGG	11	0.22
Leu1	CUU	44	0.48	Pro	CCU	61	1.81	His	CAU	62	1.59	Arg	CGU	21	1.4
	CUC	6	0.07		CCC	15	0.44		CAC	16	0.41		CGC	2	0.13
	CUA	60	0.65		CCA	57	1.69	Gln	CAA	67	1.65		CGA	32	2.13
	CUG	2	0.02		CCG	2	0.06		CAG	14	0.35		CGG	5	0.33
Ile	AUU	331	1.74	Thr	ACU	60	1.26	Asn	AAU	167	1.71	Ser1	AGU	27	0.66
	AUC	50	0.26		ACC	34	0.71		AAC	28	0.29		AGC	3	0.07
Met	AUA	242	1.72		ACA	94	1.97	Lys	AAA	53	1.47		AGA	67	1.65
	AUG	39	0.28		ACG	3	0.06		AAG	19	0.53		AGG	2	0.05
Val	GUU	81	1.87	Ala	GCU	71	1.74	Asp	GAU	67	1.74	Gly	GGU	98	1.68
	GUC	6	0.14		GCC	18	0.44		GAC	10	0.26		GGC	3	0.05
	GUA	73	1.69		GCA	70	1.72	Glu	GAA	64	1.71		GGA	115	1.97
	GUG	13	0.3		GCG	4	0.1		GAG	11	0.29		GGG	18	0.31

Supplementary Table S3. Frequency and RSCU values of codon in PCGs in the mitogenomes (cont.)

T. mitratus															
**Amino acid**	**Codon**	**Count**	**RSCU**	**Amino acid**	**Codon**	**Count**	**RSCU**	**Amino acid**	**Codon**	**Count**	**RSCU**	**Amino acid**	**Codon**	**Count**	**RSCU**
Phe	UUU	253	1.56	Ser2	UCU	92	2.1	Tyr	UAU	157	1.77	Cys	UGU	44	1.8
	UUC	71	0.44		UCC	28	0.64		UAC	20	0.23		UGC	5	0.2
Leu2	UUA	351	3.75		UCA	106	2.42	Stop	UAA (*)	-	-	Trp	UGA	91	1.8
	UUG	82	0.88		UCG	12	0.27		UAG (*)	-	-		UGG	10	0.2
Leu1	CUU	48	0.51	Pro	CCU	71	2.07	His	CAU	63	1.62	Arg	CGU	20	1.31
	CUC	15	0.16		CCC	11	0.32		CAC	15	0.38		CGC	1	0.07
	CUA	56	0.6		CCA	49	1.43	Gln	CAA	69	1.73		CGA	34	2.23
	CUG	10	0.11		CCG	6	0.18		CAG	11	0.28		CGG	6	0.39
Ile	AUU	315	1.68	Thr	ACU	59	1.24	Asn	AAU	151	1.63	Ser1	AGU	30	0.69
	AUC	60	0.32		ACC	22	0.46		AAC	34	0.37		AGC	6	0.14
Met	AUA	212	1.62		ACA	104	2.18	Lys	AAA	61	1.65		AGA	73	1.67
	AUG	49	0.38		ACG	6	0.13		AAG	13	0.35		AGG	3	0.07
Val	GUU	69	1.52	Ala	GCU	79	1.98	Asp	GAU	66	1.76	Gly	GGU	88	1.55
	GUC	10	0.22		GCC	19	0.47		GAC	9	0.24		GGC	4	0.07
	GUA	87	1.92		GCA	54	1.35	Glu	GAA	68	1.77		GGA	117	2.06
	GUG	15	0.33		GCG	8	0.2		GAG	9	0.23		GGG	18	0.32

Supplementary Table S4. Ka/Ks ratio of 13 protein-coding genes in 24 species of Gryllidae.

CDS	Ka/Ks (SLAC)	p-value (BUSTED)	Episodic adaptation
atp6	0.064	0.5	No
atp8	0.1783	0.5	No
cox1	0.0204	**0.00074**	Yes
cox2	0.047	0.17	No
cox3	0.0703	**3.3E-10**	Yes
cytb	0.0662	**0.00098**	Yes
nad1	0.084	0.29	No
nad2	0.1256	0.14	No
nad3	0.0975	0.5	No
nad4	0.1179	0.44	No
nad4L	0.0824	0.14	No
nad5	0.1232	**0.000016**	Yes
nad6	0.1601	0.46	No

Supplementary Table S5. Comparative analysis of the secondary structures of 22 tRNAs between *G. bimaculatus* and *T. mitratus.*

tRNA type	*G. bimaculatus*	*T. mitratus*	Structural comparison
trnA, trnR, trnN	Standard cloverleaf; long T-stems.	Reduced T-stems; smaller D-loops.	*T. mitratus* shows minor truncation in lateral arms.
trnD, trnC, trnQ	Well-defined stems and loops.	Reduced T-arm in *trnQ*.	*T. mitratus trnQ* is more compact than *G. bimaculatus*.
trnE, trnG, trnH	Robust D-arms with 3-4 base pairs.	Truncated D-arms; smaller loops.	*T. mitratus* displays genome compaction in these units.
trnI, trnL1, trnL2	Standard; trnL2 has large T-loop.	trnL2 T-loop is significantly smaller.	*T. mitratus* shows loss of non-essential nucleotides.
trnK, trnM, trnF	Symmetrical cloverleaf structure.	Mostly identical to *G. bimaculatus*.	Highly conserved structures in both species.
trnP	Full 4-arm structure.	Shortened D-arm.	*T. mitratus* exhibits asymmetrical reduction.
trnS1 (GCU)	Lacks D-arm (DHU arm).	Lacks D-arm (DHU arm).	Common feature in Orthoptera mitogenomes.
trnS2 (UGA)	Complete cloverleaf.	Severely reduced D-arm.	*T. mitratus trnS2* is transitioning toward truncation.
trnT, trnW	Standard arm lengths.	Significant shorter T-arms.	Extreme truncation observed in *T. mitratus*.
trnY, trnV	Stable 4-arm cloverleaf.	Slightly shorter acceptor stems.	Minor variations in stem length.

Supplementary Table S6. Results of best fit models for each alignment partition by PartitonFinder2

**alignment**	.\concatenation.phy
**branch lengths**	linked
**models**	JC, K80, SYM, F81, HKY, GTR, JC+G, K80+G, SYM+G, F81+G, HKY+G, GTR+G, JC+I, K80+I, SYM+I, F81+I, HKY+I, GTR+I, JC+I+G, K80+I+G, SYM+I+G, F81+I+G, HKY+I+G, GTR+I+G
**model selection**	aic
**search**	greedy
	**Best partitioning scheme**
**Scheme lnL**	-160358.2128
**Scheme AICc**	321230.4256
**Number of params**	257
**Number of sites**	15994
**Number of subsets**	21

Subset	Best Model	# sites	Partition names
**1**	GTR+I+G	681	atp6_mafft
**2**	GTR+I+G	759	atp8_mafft nad6_mafft
**3**	GTR+I+G	1545	cox1_mafft
**4**	GTR+I+G	1485	cox3_mafft cox2_mafft
**5**	GTR+I+G	1146	cytb_mafft
**6**	GTR+I+G	1275	nad4L_mafft nad1_mafft
**7**	GTR+I+G	1110	nad2_mafft
**8**	GTR+G	372	nad3_mafft
**9**	GTR+I+G	3264	nad4_mafft nad5_mafft
**10**	GTR+I+G	1614	rrnL_mafft
**11**	GTR+G	1028	rrnS_mafft
**12**	GTR+I+G	363	trnK_mafft, trnL2_mafft, trnT_mafft, trnA_mafft, trnN_mafft
**13**	GTR+I+G	221	trnP_mafft, trnC_mafft, trnE_mafft
**14**	HKY+I+G	72	trnD_mafft
**15**	GTR+G	316	trnH_mafft, trnF_mafft, trnY_mafft, trnW_mafft
**16**	HKY+I+G	73	trnG_mafft
**17**	GTR+G	159	trnI_mafft, trnM_mafft
**18**	HKY+G	86	trnL1_mafft
**19**	HKY+G	84	trnQ_mafft
**20**	GTR+G	192	trnR_mafft, trnS2_mafft
**21**	GTR+G	149	trnS1_mafft, trnV_mafft
