# Supplementary figures and images for: Complete Mitochondrial Genomes and Evolutionary Insights of Two Commercially Farmed Edible Crickets (Gryllus bimaculatus and Teleogryllus mitratus) from Thailand
Source: Animals (Basel). 2026 Apr 23;16(9):1305. doi: 10.3390/ani16091305 (PMC13163110; doi:10.3390/ani16091305)

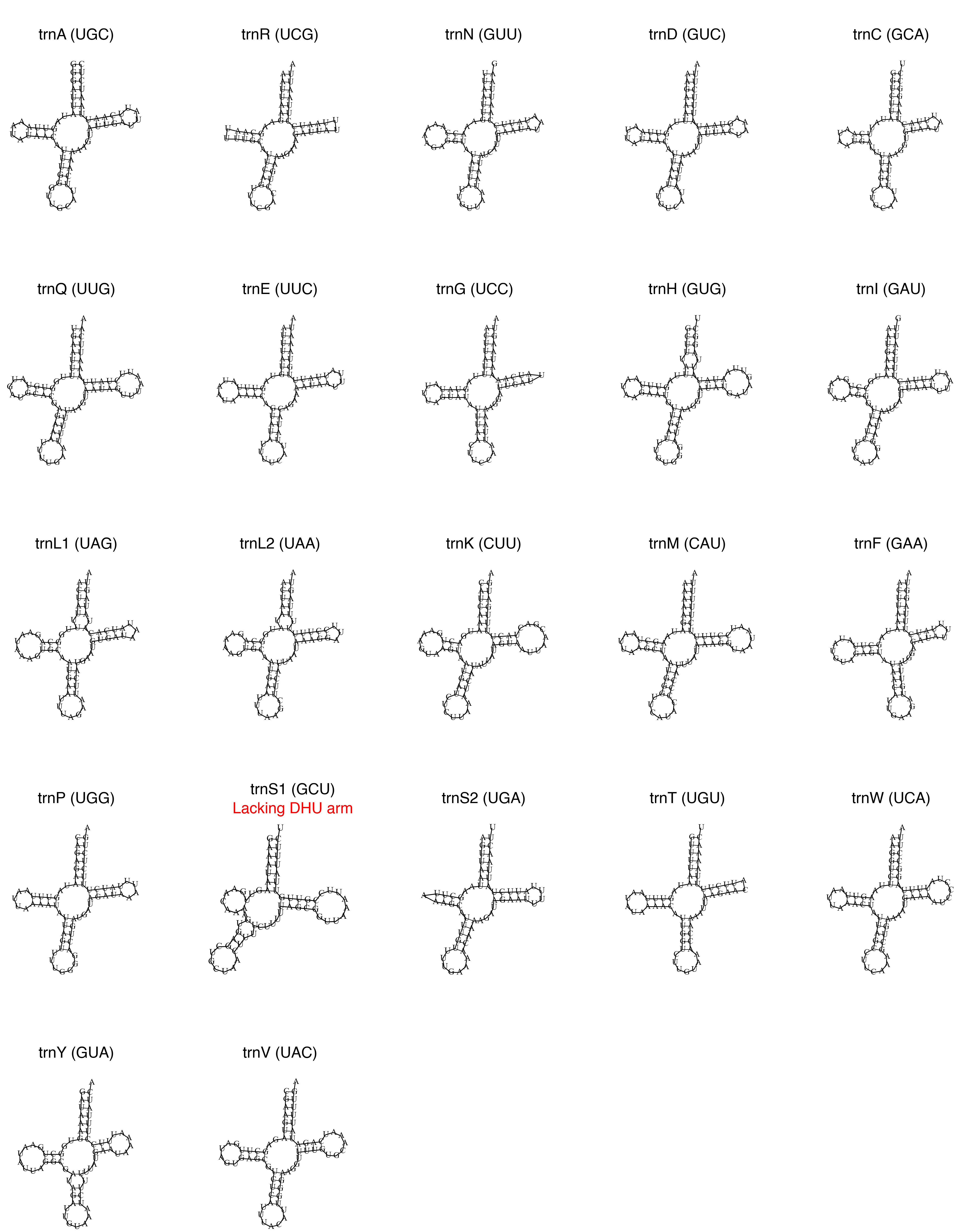

Supplement: Supplementary file 1 [file animals-16-01305-s001.zip › animals-4236102-supplementary/Figure S1.png]

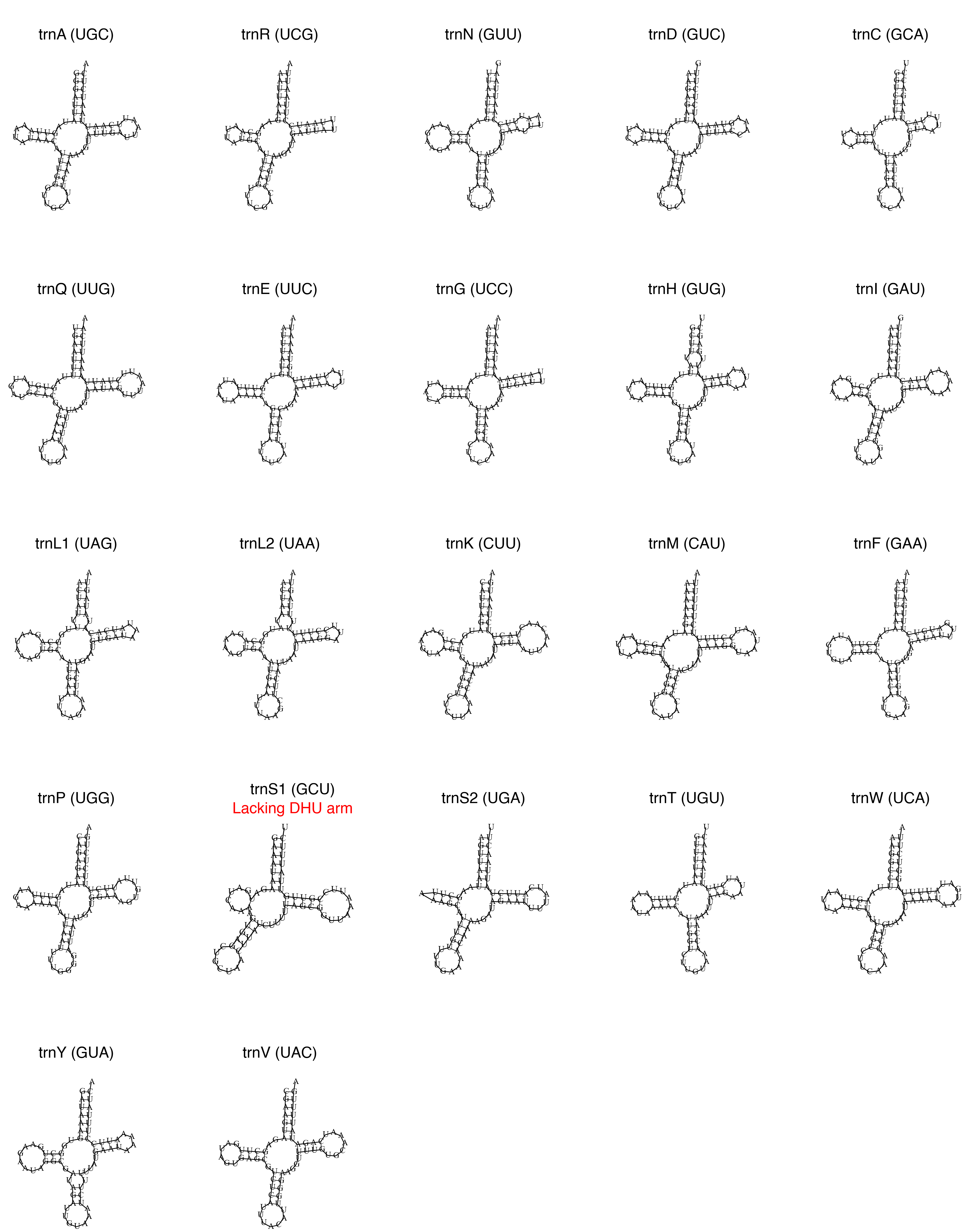

Supplement: Supplementary file 1 [file animals-16-01305-s001.zip › animals-4236102-supplementary/Figure S2.png]
